# Peroxisomal ABCD1 deficiency in mice drives Th1 bias through 25-HC–LXR signaling in CD4^+^ T cells

**DOI:** 10.3389/fimmu.2026.1722647

**Published:** 2026-05-20

**Authors:** Reina Maeda, Mayu Oishi, Ryutaro Nagamori, Mari Hikosaka-Kuniishi, Shiro Watanabe, Jung-Bum Lee, Masashi Morita, Takanori So

**Affiliations:** 1 Laboratory of Molecular Cell Biology, Graduate School of Medicine and Pharmaceutical Sciences, University of Toyama, Toyama, Japan; 2 Department of Technical Assistance, Research Promotion Organization, University of Toyama, Toyama, Japan; 3 Laboratory of Plant Resource Sciences, Graduate School of Medicine and Pharmaceutical Sciences, University of Toyama, Toyama, Japan

**Keywords:** 25-hydroxycholesterol, ABCD1, Blimp-1, CD4+ T cell, LXR, peroxisome, X-linked adrenoleukodystrophy

## Abstract

X-linked adrenoleukodystrophy (X-ALD) is driven by ABCD1 dysfunction, causing very-long-chain fatty acid (VLCFA) accumulation and cerebral inflammation, yet the role of T cells in X-ALD remains unclear. Here, we show that *Abcd1*-deficient CD4^+^ T cells exhibit a strong Th1 bias, producing more IFN-γ and less IL-10 under antigen-specific immunization *in vivo* and Th1-polarizing conditions *in vitro*. Transcriptional profiling revealed early induction of *Ifng* and *Tbx21* (T-bet) and late repression of *Prdm1* (Blimp-1), indicating Blimp-1–dependent derepression of IFN-γ and reduced IL-10. Mechanistically, liver X receptor (LXR) signaling was markedly amplified, evidenced by upregulation of *Abca1*, *Srebf1*, and the oxysterol 25-hydroxycholesterol (25-HC), driven by increased *Ch25h*. Pharmacological modulation validated this axis: the LXR antagonist SR9238 restored Blimp-1 and IL-10 while reducing IFN-γ, whereas the LXR agonist T0901317 and exogenous 25-HC recapitulated the *Abcd1*-deficient phenotype. Thus, 25-HC–LXR signaling suppresses Blimp-1, enforcing Th1 polarization in *Abcd1*-deficient CD4^+^ T cells. These findings define an immunometabolic link between peroxisomal lipid metabolism and T cell differentiation and highlight the 25-HC–LXR–Blimp-1 axis as a mechanistic link regulating CD4^+^ T−cell polarization, with potential relevance to X-ALD–associated neuroinflammation.

## Introduction

X-linked adrenoleukodystrophy (X-ALD) is an inherited peroxisomal lipid-metabolism disorder caused by loss-of-function mutations in the *ABCD1* gene. In the most severe cerebral form of X-ALD, a hallmark of the disease is inflammatory demyelination with axonal degeneration in the central nervous system (CNS) (1–3). Despite intensive study, no effective disease-modifying pharmacological therapy exists for X-ALD, largely because the molecular mechanisms that initiate and amplify cerebral inflammation remain incompletely defined (4–6).

ABCD1 encodes a peroxisomal transporter required for the import of very-long-chain fatty acyl-CoAs (VLCFA-CoAs) into peroxisomes (7, 8). ABCD1 deficiency causes accumulation of VLCFAs in gangliosides, phospholipids, and cholesterol esters within cerebral white matter. However, lipid accumulation alone is insufficient to induce inflammatory demyelination. Prior work has linked VLCFA-driven oxidative stress to innate and adaptive immune activation (9–11), and additional factors—such as genetic modifiers and environmental triggers including head trauma or viral infection—have been proposed to contribute to disease onset and progression (12–15). Nevertheless, how cerebral inflammation is initiated and propagated in X-ALD remains unclear.

Astrocytes, microglia, and macrophages are implicated in inflammatory demyelination in X-ALD (16–18). By contrast, the contribution of T lymphocytes—particularly CD4^+^ T cells—has received less attention. Antigen-presenting cells (APCs) expressing CD1 molecules have been identified in early white matter lesions of X-ALD (19), and CD8^+^ T cells are thought to participate in the initial phases of demyelination (19–21). Disruption of blood-brain barrier (BBB) precedes demyelination, and areas of BBB breakdown are infiltrated CD8^+^ and CD4^+^ T cells (22, 23). Although CD4^+^ T cells are not consistently abundant in ALD lesions (19, 22), their central role in orchestrating adaptive immunity through cytokine secretion and cell–cell interactions make them compelling candidates in X-ALD pathogenesis.

Because ABCD1 regulates peroxisomal lipid metabolism, the loss of ABCD1 may alter CD4^+^ T cell polarization and effector functions—for example, by skewing differentiation toward IFN-γ-producing Th1 cells (24)—with potential implications for neuroinflammation and demyelination. Although *Abcd1*-knockout mice do not develop overt cerebral pathology, they instead display an adrenomyeloneuropathy (AMN)-like or preclinical X-ALD phenotype (25). Therefore, determining whether *Abcd1*-deficient CD4^+^ T cells acquire altered functional properties is crucial for understanding their potential role as active drivers of inflammation and for guiding the development of targeted immunotherapies.

Here, we demonstrate that *Abcd1*-deficiency in mice markedly alters CD4^+^ T cell function, leading to increased IFN-γ production. Mechanistically, our data support a model in which 25-hydroxycholesterol (25-HC)–dependent activation of liver X receptor (LXR) signaling suppresses the transcriptional repressor Blimp-1, thereby derepressing IFN-γ and diminishing IL-10, culminating in a Th1-skewed response. These findings reveal an immunometabolic link between peroxisomal lipid metabolism and adaptive immunity that may be relevant to inflammatory processes associated with X-ALD.

## Materials and methods

### Mice

The *Abcd1*-deficient mouse line (B6Cg-Abcd1<​​​tmYmd>​​​) was originally generated by Kobayashi et al. (26). These mice were backcrossed to the C57BL/6J background (Japan SLC, Inc.) for 10 generations. Heterozygous *Abcd1*^+/-^ C57BL/6J mice were intercrossed to generate *Abcd1*^+/+^ (wild-type, WT) and *Abcd1*^-/-^ (*Abcd1*-deficient, knockout, KO) mice. Wild-type and *Abcd1*-deficient male mice aged 8–12 weeks were provided food and water *ad libitum*. *Abcd1*-deficient mice develop a late-onset axonopathy resembling the AMN phenotype, but do not exhibit cerebral inflammatory demyelination characteristic of cerebral ALD (CALD), as previously reported (25). Therefore, this model reflects an X-ALD–like/AMN–like condition rather than a bona fide CALD phenotype. All animals were bred and maintained under specific pathogen-free conditions, and all experimental procedures were conducted in accordance with protocols approved by the Animal Care and Use Committee of the University of Toyama (Approval No. A2022PHA-11).

### Antibodies and cytokines

Anti-CD3ε (low endotoxin, azide-free; 145-2C11, 100340), anti-CD28 (low endotoxin, azide-free; 37.51, 102116), anti-IL-4 (low endotoxin, azide-free; 11B11, 504122), as well as fluorescein isothiocyanate (FITC)-anti-CD4 (RM4-5, 100510), phycoerythrin (PE)-anti-CD62L (MEL-14; 104407), allophycocyanin (APC)-anti-CD44 (IM7, 103011), APC-anti-IFN-γ (XMG1.2, 505809), PE-anti-CD8α (53-6.7, 100707), and PE/Cyanine7-anti-mouse CD4 (clone GK1.5, 100421) antibodies were obtained from BioLegend (San Diego, CA, U.S.A.). Isotype control antibodies, including FITC-Rat IgG1, κ (400405), PE-Rat IgG1, κ (400408), APC-Rat IgG1, κ (400412), were also purchased from BioLegend. Recombinant mouse IL-12 (095–05331), TGF-β1 (209–16544), and IL-6 (099-04631) were obtained from Fujifilm Wako (Osaka, Japan), and IL-4 (214-14) was purchased from PeproTech (Cranbury, NJ, U.S.A).

### Preparation of CD4^+^ T cells and *in vitro* T cell differentiation

Spleen cells were prepared from 2- to 3-month-old male mice in Hanks’ Balanced Salt Solution (HBSS) by passing the tissue through a 200-µm stainless steel mesh, followed by filtration through a 40-µm mesh to obtained single-cell suspension. Cells were pelleted by centrifugation, and after erythrocyte lysis with Gey’s balanced salt solution, naïve CD4^+^ T cells were isolated by negative selection using immunomagnetic beads (Naïve CD4^+^ T cell Isolation Kit, 130-104-453, Miltenyi Biotec, Bergisch Gladbach, Germany) according to the manufacturer’s instructions. Total CD4^+^ T cells were isolated by positive selection using immunomagnetic beads (CD4 [L3T4] MicroBeads, 130-117-043, Miltenyi Biotec). Cell purity was assessed by fluorescence-activated cell sorting (FACS) and consistently exceeded 90%.

Isolated cells were cultured in RPMI1640 (Fujifilm Wako) supplemented with 10% fetal bovine serum, 100 U/ml penicillin, 100 μg/ml streptomycin, and 50 μM 2-mercaptoethanol, hereafter referred to as complete medium. Naïve CD4^+^ T cells (1 x 10^5^) were plated in 96-well culture plates and stimulated for the indicated time with plate-bound anti-CD3ε antibody (3 µg/ml) and soluble anti-CD28 antibody (1 μg/ml) in the presence of recombinant mouse IL-12 (10 ng/ml) and anti-mouse IL-4 antibody (5 µg/ml) for Th1 differentiation, IL-4 (10 ng/ml) and anti-mouse IFN-γ antibody (5 µg/ml) for Th2 differentiation, or TGF-β1 (10 ng/ml) and IL-6 (10 ng/ml) for Th17 differentiation.

### Flow cytometry

The purity of naïve CD4^+^ T cells was assessed by staining with cell surface markers. Splenocytes or naïve CD4^+^ T cells from wild-type and *Abcd1*-deficient mice were washed with FACS buffer (0.2% bovine serum albumin (BSA) and 0.02% NaN_3_ in phosphate-buffered saline (PBS)) and incubated with antibodies (FITC-anti-CD4, PE-anti-CD62L, or APC-anti-CD44) for 30 minutes on ice. Cell population analysis of draining lymph nodes was performed by staining with FITC-anti-CD4, PE-anti-CD8α, PE-anti-CD62L, and APC-anti-CD44 antibodies, as described above. Data were acquired using a FACSCanto II flow cytometer (BD Biosciences, Franklin Lakes, NJ, U.S.A) and analyzed with Flowing software 2.5.1 (Turku Bioscience, Turku, Finland; available at http://flowingsoftware.btk.fi/, accessed on 19 September 2021).

### Intracellular staining

For detect intracellular IFN-γ, stimulated CD4^+^ T cells were reactivated with 50 ng/ml phorbol 12-myristate 13-acetate (Fujifilm Wako) and 1 μg/ml ionomycin (Cayman Chemical, Michigan, U.S.A.) in the presence of 2 μM monensin (BioLegend, 420701) to inhibit cytokine secretion. After a 5-hour incubation, cells were stained extracellularly with a FITC-anti-CD4 antibody on ice for 15 min, fixed with 2% paraformaldehyde for 20 min, and permeabilized using buffer solutions (BioLegend 421002). Following blocking with Rat IgG1, cells were stained intracellularly with an APC-IFN-γ antibody or an APC-isotype control IgG antibody diluted in permeabilization buffer. Intracellular IFN-γ levels were quantified by measuring the mean fluorescence intensity (MFI) of IFN-γ-positive cells. Data were acquired using a BD FACSCanto II flow cytometer (BD Biosciences) and analyzed with Flowing Software.

### Real-time PCR

Total RNA was extracted using Isogen II (Nippon Gene Co., Ltd., Tokyo, Japan) and reverse-transcribed with ReverTra Ace qPCR RT Master Mix (Toyobo Co., Ltd. Osaka, Japan) according to the manufacturer’s instructions. Quantitative PCR and melting curve analyses were performed using a Brilliant SYBR Green QRT-PCR Kit (Toyobo Co., Ltd.) on an Mx3005P QPCR system (Agilent Technologies, Santa Clara, CA, U.S.A.). PCR conditions consisted of an initial denaturation at 95˚C for 3 minutes, followed by 40 cycles of 95˚C for 15 seconds and 60˚C for 60 seconds. Relative gene expression levels were calculated using the comparative Ct method and normalized to *Ppib* mRNA. Results for each gene are expressed relative to the control, which was arbitrarily set to 1. Data are presented as mean ± SD. Primer sequences used in this study are listed in **Supplementary Table 1**.

### ELISA

Cytokine concentrations in culture supernatants were measured using a sandwich enzyme-linked immunosorbent assay (ELISA). Briefly, 96-well enzyme immunoassay plates (Thermo Fisher, Waltham, MA, U.S.A.) were coated overnight at 4 °C with purified capture antibodies. After washing with PBS containing 0.05% Tween-20 and blocking with 0.5% BSA in PBS for 1 h, samples and standards were added to the plates and incubated for 2 hours. Subsequently, biotin-labeled anti-mouse detection antibodies were added and incubated for 1 hour. Bound cytokines were detected using a streptavidin-HRP (BioLegend), and absorbance at 450 nm was measured using a FilterMaxF5 microplate reader (Molecular Devices, San Jose, CA, U.S.A.). Capture and detection antibodies were purchased from BioLegend as follows: anti-IFN-γ (R4-6A2, 505702) capture antibody, biotin-anti-IFN-γ (XMG1.2, 505804) detection antibody; anti-mouse IL-10 (JES5-16E3, 505001) capture antibody, biotin-anti-IL-10 (JES5-2A5, 504905) detection antibody; anti-IL-17A (TC11-18H10.1, 506901) capture antibody, biotin-anti-IL-17A (TC11-8H4, 507001) detection antibody; anti-IL-4 (11B11, 504101) capture antibody, biotin-anti-IL-4 (BVD6-24G2, 504201) detection antibody. HRP-conjugated streptavidin (405210), and 3,3’,5,5’-tetramethylbenzidine substrate (421101) were also obtained from BioLegend.

### T cell restimulation assay

*Abcd1*-deficient mice and wild-type mice aged between 8–12 weeks were immunized subcutaneously in the footpad with 50 μg of ovalbumin (OVA) emulsified in complete Freund’s adjuvant (CFA, F5881, Merck) on day 0. The emulsion was prepared by mixing equal volumes of ovalbumin in PBS and CFA. One week later, cells from draining lymph nodes were isolated and plated in 96-well plates. The cells were restimulated with ovalbumin (0, 0.1, 1, or 10 μM) in complete medium, and culture supernatants were collected after 96 hours for cytokine ELISA. Cell proliferation was assessed using the MTT assay.

### GC-MS analysis of 25-HC

Quantification of 25-hydroxycholesterol (25-HC) in the medium was performed using gas chromatography-mass spectrometry (GC-MS). Lipids were extracted from the medium using the Bligh and Dyer method. A deuterium-labeled internal standards (1 μg of D_6_-25OHC, Cayman Chemical) was added to the medium prior to extraction. After solvent evaporation, lipid extracts were dissolved in n-hexane and applied to solid phase extraction cartridges (Bondesil-SI 40 μm, 100 mg, Agilent Technologies, Santa Clara, CA, U.S.A.). Sterols were eluted with ethyl acetate and dried. The residue was dissolved in 10 μL of pyridine and 50 μl of *N,O*-bis(trimethylsilyl)trifluoroacetamide (BSTFA) plus trimethylchlorosilane (TMCS) (Tokyo Kasei, Tokyo, Japan), then incubated at 40 °C for 30 min for trimethylsilylation.

GC-MS analysis was carried out using a Shimadzu GCMS-QP2010 Ultra system (Shimadzu, Kyoto, Japan) equipped with a DB-5MS column (30 m × 0.32 mm × 0.25 μm film thickness; Agilent Technologies, Santa Clara, CA, U.S.A.) in 70-eV electron ionization mode. The oven temperature program was as follows: 60 °C for 3 min, then increased at 15 °C/min to 310 °C, and held for 10 minutes. Helium was used as the carrier gas at a constant linear velocity of 50.0 cm/s. One microliter of sample was injected at an injector temperature of 250 °C, and the MS interface temperature was maintained at 280 °C. Quantification was performed using selected ion monitoring (SIM) by recording ions at m/z 456.40 for the 25-hydroxycholesterol-trimethylsilyl derivative and m/z 462.40 for D_6_-25OHC.

### VLCFA analysis

VLCFA analysis of total CD4^+^ T cells was performed as previously described (17). Total lipids extracted using the Bligh and Dyer method were treated with methanol containing 5% (w/v) hydrogen chloride at 100˚C for 2 hours to prepare fatty acid methyl esters. The fatty acid methyl esters were purified using a solid-phase extraction cartridge (InterSep SI, GL Sciences, Tokyo, Japan) and analyzed by gas-liquid chromatography with a capillary column (DB-225MS, Agilent Technologies). The C22:0 and C26:0 methyl esters were identified by comparison with authentic standards (Sigma, St. Louis. MO, U.S.A.). Data are represented as the amount of C26:0 per milligram of cellular protein (μg/mg protein).

### Fatty acid β-oxidation

β-Oxidation was measured as previously described (27). Total CD4^+^ T cells were suspended in 0.25 M sucrose containing 1 mM EDTA and 10 mM Tris-HCl (pH 8.0) and incubated with [1-^14^C]lignoceric acid (53 mCi/mmol; Moravek Biochemicals, Brea, CA, U.S.A.), which had been solubilized in 0.05 mL of α-cyclodextrin (10 mg/ml) in 10 mM Tris-HCl (pH 8.0), for 60 minutes at 37 °C. Reactions were terminated by adding 0.05 mL of 1 N KOH and perchloric acid to a final concentration of 6%, and samples were kept at 4 °C overnight. After centrifugation, the supernatant was subjected to Folch partitioning, and the aqueous phase was collected for scintillation counting.

### Immunoblotting

Immunoblotting was performed using the Immobilon Classico Western HRP substrate reagent (Merck KGaA, Darmstadt, Germany) as previously described (28). A rabbit anti-ABCD3 antibody raised against the COOH-terminal 15 amino acids of rat Abcd3 (29) and a rabbit anti-ABCD1 antibody raised against the COOH terminal 24 amino acids of human ABCD1 (28) were used in this study. Additional antibodies were obtained from commercial sources, including a polyclonal rabbit anti-ABCD2 antibody (ABclonal, Inc., Tokyo, Japan), a monoclonal mouse anti-GAPDH antibody (Fujifilm Wako), an a monoclonal rat anti-Blimp-1 antibody (Santa Cruz Biotechnology, Inc., Dallas, TX, U.S.A.), HRP-conjugated Affinipure Goat anti-Rat IgG(H+L) (Proteintech, Rosemont, IL, U.S.A.), and HRP-conjugated anti-rabbit IgG (H+L) and anti-mouse IgG (H+L) antibodies (MBL Life Science, Aichi, Japan).

### MTT assay

Cell proliferation was assessed using the MTT assay with a 3-(4,5-dimethylthiazol-2-yl)-2,5-diphenyl-tetrazolium bromide (MTT; M2128, Millipore Sigma, Burlington, MA, U.S.A.). The water-insoluble MTT formazan was solubilized in dimethyl sulfoxide (DMSO), and the absorbance was measured with a plate reader at 570 nm using a FilterMax F5 microplate reader (Molecular Devices). Protein concentration was determined by the Lowry method using BSA as the standard (30).

### Statistical analysis

Statistical analyses were performed using Student’s two-tailed unpaired t-test for comparisons between two groups, or one-way ANOVA followed by Dunnett’s *post-hoc* test for multiple comparisons against a single control. In experiments involving two independent variables, statistical significance was assessed using two-way ANOVA with genotype and dose or time as factors. Following a significant interaction, *post hoc* comparisons were conducted with Holm correction for multiple testing. All experiments were independently repeated at least twice with similar results unless otherwise noted. Data are presented as mean ± SEM. Statistical significance was defined as *p* < 0.05 and is indicated in figures as *p* < 0.05 (*), *p* < 0.01 (**), and *p* < 0.001 (***).

## Results

### Enhanced antigen-specific Th1-type response in *Abcd1*-deficient mice

**Figure 1:**
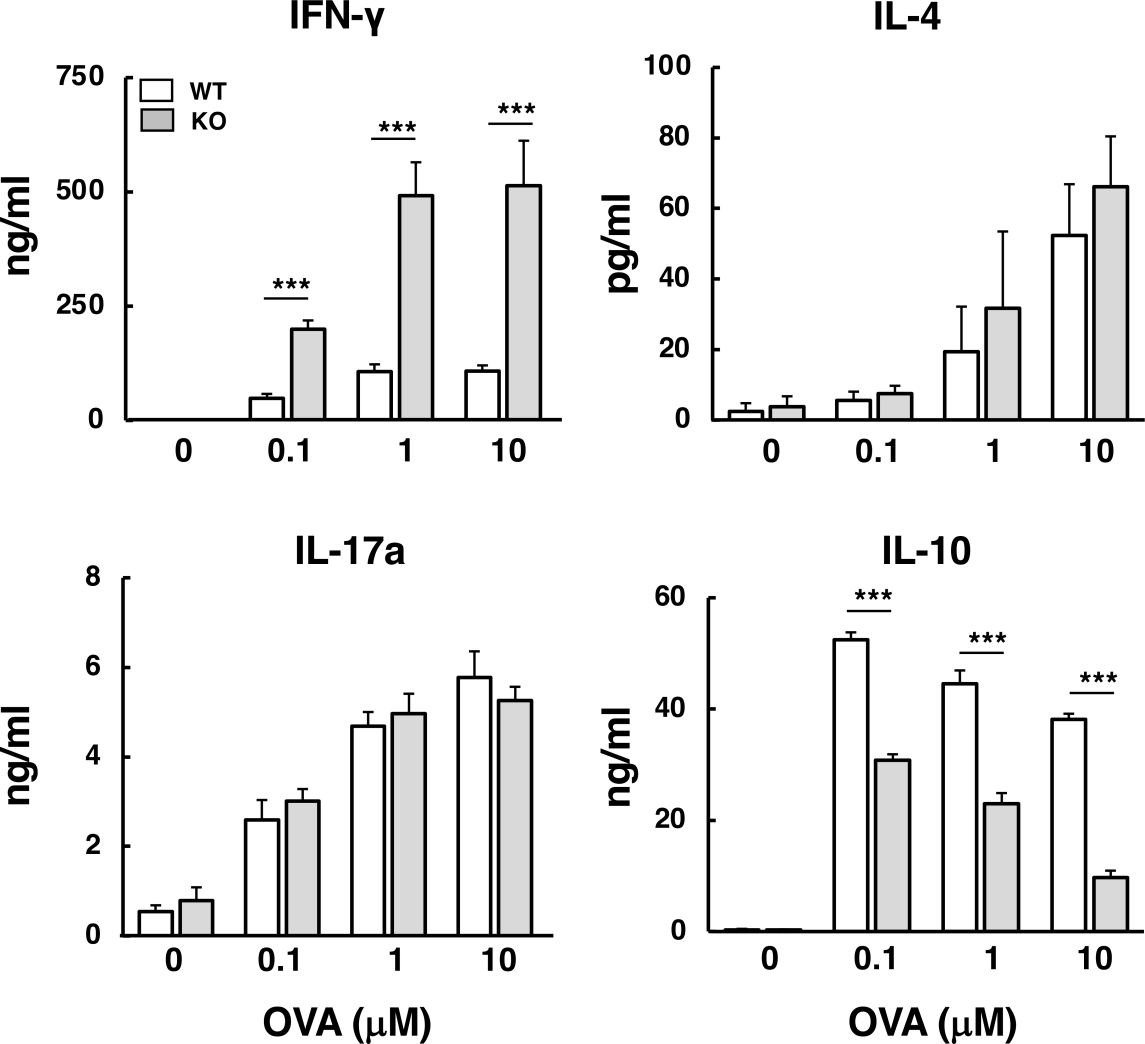
Antigen-specific Th1-type responses in *Abcd1*-deficient lymph node cells. Wild-type (WT) and *Abcd1*-deficient (knockout; KO) mice were immunized in the footpads with ovalbumin (OVA) emulsified in complete Freund’s adjuvant (CFA). Two weeks later, cells from draining lymph nodes (dLNs) were cultured and re-stimulated *ex vivo* with OVA (0, 0.1, 1, or 10 µM) for 4 days. Cytokine concentrations in culture supernatants—IFN-γ, IL-10, IL-4, and IL-17A—were quantified by ELISA. Bars: white, WT; gray, KO. Data are mean ± SD (n = 5 mice per group). Statistically significant differences between WT and KO groups were analyzed by two-way ANOVA with Holm-corrected *post hoc* tests (***p< 0.001).

To determine whether *Abcd1* deficiency influences antigen-specific T cell responses, wild-type and *Abcd1*-deficient mice were immunized with OVA antigen emulsified in CFA. Notably, OVA/CFA immunization was employed as a robust and standardized peripheral stimulus to evaluate T cell responsiveness, rather than to model spontaneous CALD-like inflammation. Two weeks later, draining lymph nodes from both groups were restimulated with OVA *ex vivo*. Flow cytometric analysis revealed no significant difference in the frequencies of total CD8^+^ cells, total CD4^+^cells, or CD4^+^ T cell subset (including naïve, effector, and memory CD4^+^ T cells) between wild-type and *Abcd1*-deficient mice (**Supplementary Figure 1**). Interestingly, upon OVA restimulation, culture supernatants from *Abcd1*-deficient cells contained significantly higher levels of IFN-γ than those from wild-type cells at all antigen concentrations (**Figure 1**). Conversely, IL-10 production was significantly lower in *Abcd1*-deficient cells, whereas IL-4 and IL-17 production did not differ between the groups. Under the same conditions, proliferative responses were not significantly different between the groups at higher concentrations (1 and 10 µM) (**Supplementary Figure 2**). Collectively, these results indicate that *Abcd1*-deficient CD4^+^ T cells display enhanced Th1-type responses under peripheral antigenic stimulation.

### Impaired VLCFA metabolism in *Abcd1*-deficient CD4^+^ T cells

**Figure 2:**
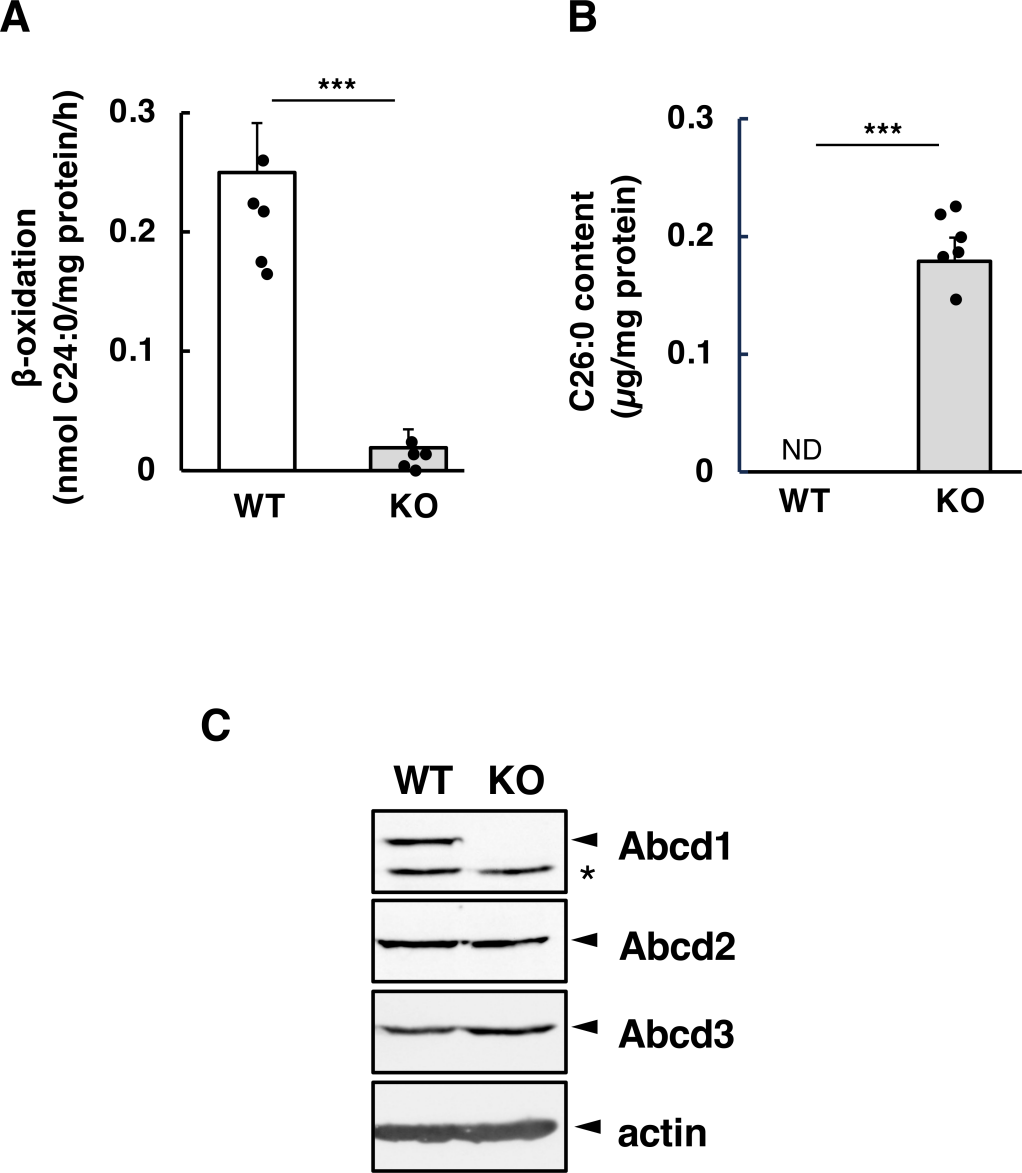
VLCFA metabolism and expression of peroxisomal ABC transporters in *Abcd1*-deficient CD4^+^ T cells. Total CD4^+^ T cells were isolated from the spleens of wild-type (WT) and *Abcd1*-deficient (KO) mice, activated with anti-CD3/anti-CD28 antibodies for 72 h, and then cultured in IL-2–containing medium for an additional 72 h. **(A)** VLCFA β-oxidation activity was measured using [1-^14^C]C24:0 as substrate. Activities for WT (white bars) and KO (gray bars) cells are expressed as nmol·mg^-^¹·h^-^¹ (mean ± SD; n = 5). ***p < 0.001 compared with WT (Student’s *t*-test). **(B)** VLCFA content was determined by gas–liquid chromatography. Values for WT (white bars) and KO (gray bars) cells are expressed as C26:0 (μg per mg cellular protein) (mean ± SD; n = 6). ***p < 0.001 compared with WT (Student’s *t*-test). **(C)** Total CD4^+^ T cells before and after activation were harvested and solubilized in sample buffer. Equal amounts of protein (150 μg per lane) were separated by SDS-PAGE and analyzed by immunoblotting for peroxisomal ABC transporters (targets indicated). The asterisk denotes a non-specific band.

Given that antigen-specific lymph node responses depend on antigen-primed CD4^+^ T cells, the enhanced Th1-type response may result from altered activity of this cell type. First, we examined whether the absence of Abcd1 leads to impaired VLCFA metabolism in CD4^+^ T cells, specifically reduced C24:0 β-oxidation activity and increased C26:0 levels. Cell population analysis indicated that both groups of unimmunized mice had similar CD4^+^ T cell subsets, including naïve, effector, and memory CD4^+^ cells, in the spleen (**Supplementary Figure 3**). As expected, when CD4^+^ T cells were activated with anti-CD3/CD28 antibodies in the presence of IL-2, C24:0 β-oxidation activities were significantly lower in the *Abcd1*-deficient CD4^+^ T cells than in the wild-type CD4^+^ T cells (**Figure 2A**), and a marked accumulation of C26:0 (0.18 µg/mg protein) was observed in *Abcd1*-deficient CD4^+^ T cells (**Figure 2B**). Immunoblot analysis revealed no difference in the expression levels of the major peroxisomal membrane protein Abcd3 and Abcd2, which exhibits functional redundancy with Abcd1, between wild-type and *Abcd1*-deficient CD4^+^ T cells (**Figure 2C**). These results suggest that *Abcd1*-deficient CD4^+^ T cells activated with antigen exhibit impaired VLCFA metabolism, a biochemical hallmark of X-ALD.

### Higher IFN-γproduction in *Abcd1*-deficient CD4^+^ T cells under Th1-polarization conditions

**Figure 3:**
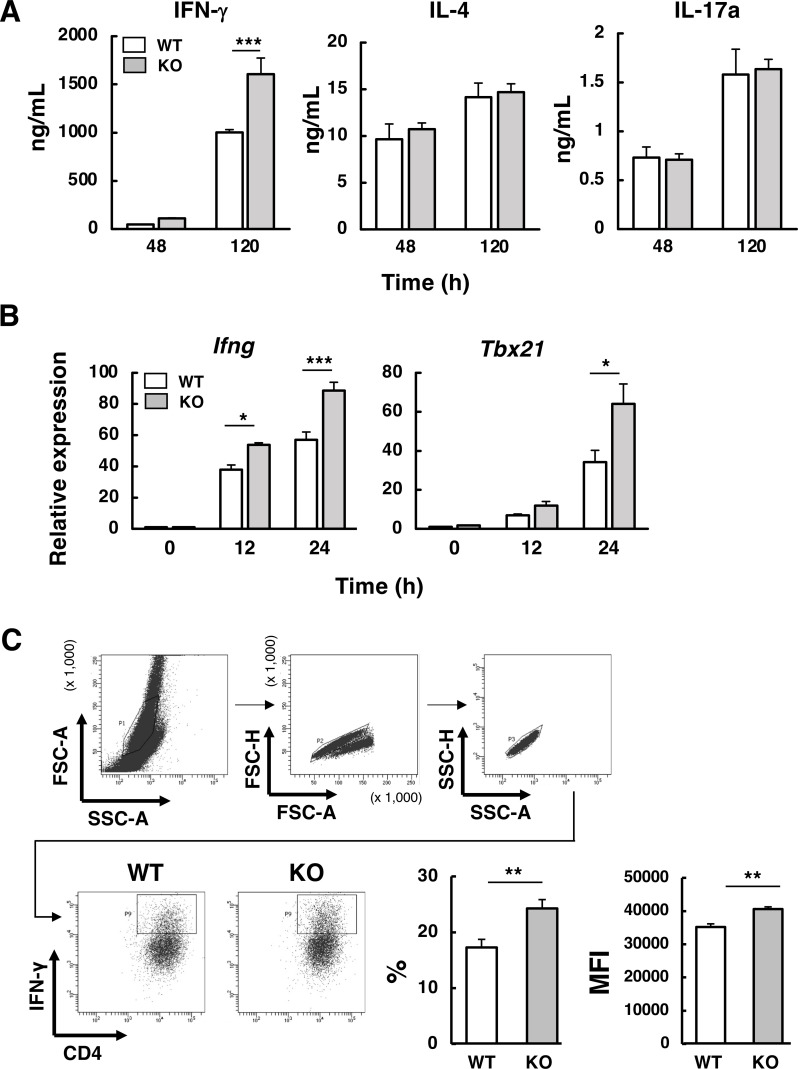
Cytokine production under Th1 polarizing conditions. **(A)** Naïve CD4^+^ T cells were isolated from the spleens of wild-type (WT) and *Abcd1*-deficient (KO) mice and activated with anti-CD3/anti-CD28 antibodies under Th1 (IL-12), Th2 (IL-4), or Th17 (IL-6 + TGF β) conditions. At 48 h and 120 h, IFN-γ, IL-4, and IL-17A concentrations in culture supernatants were quantified by ELISA. Bars: white, WT; gray, KO. Data are mean ± SD (n = 3). ***p < 0.001 compared with WT (two-way ANOVA). **(B)** Naïve CD4^+^ T cells were activated with anti-CD3/anti-CD28 antibodies in the presence of IL-12 and an anti-IL-4 neutralizing antibody for 12 h or 24 h. Total RNA was extracted and reverse transcribed for qPCR. Expression of *Ifng* and *Tbx21* was normalized to *Ppib* and expressed relative to WT at 0 h (set to 1). Bars: white, WT; gray, KO. Data are mean ± SD (n = 3). *p < 0.05, ***p < 0.001 compared with WT (two-way ANOVA). **(C)** Naïve CD4^+^ T cells were activated as in **(B)** for 48 h, then restimulated for 5 h with phorbol-12-myristate-13-acetate (PMA, 50 ng/mL) and ionomycin (1 µg/mL) in the presence of monensin (2 µM). After surface CD4 staining, cells were fixed and permeabilized, followed by intracellular staining with PE-conjugated anti-IFN-γ antibody. Flow cytometry data were acquired on a FACSCanto II (BD Biosciences) and analyzed with Flowing Software. A representative FACS plot was shown. Data show the percentage of IFN-γ–positive cells among CD4^+^ T cells and the mean fluorescence intensity (MFI) of IFN-γ. **p < 0.01 compared with WT (Student’s *t*-test).

Since antigen-specific CD4^+^ T cell–derived Th1-type responses were higher in *Abcd1*-deficient mice following peripheral immunization (**Figure 1**), we next investigated whether *Abcd1* deficiency affects differentiation into Th1, Th2, and Th17 effector T cell subsets *in vitro*. Splenic naïve CD4^+^ T cells from wild-type and *Abcd1*-deficient mouse were stimulated with anti-CD3/CD28 antibodies in the presence of cytokines milieus required for Th differentiation: IL-12 for Th1, IL-4 for Th2, or TGFβ and IL-6 for Th17. Under Th1-polarizing conditions, IFN-γ production was comparable between wild-type and *Abcd1*-deficient CD4^+^ T cells at 48 h; however, after 120 h of culture, *Abcd1*-deficient CD4^+^ T cells produced significantly higher levels of IFN-γ (**Figure 3A**). In contrast, under Th2- and Th17-polarization conditions, production of IL-4 and IL-17, respectively, did not differ between the two groups at either time point. These results suggest that Abcd1 limits Th1 differentiation but does not affect Th2 or Th17 differentiation. Because T-bet (Tbx21) is the master transcription factor driving Th1-lineage commitment and IFN-γ expression, we quantified early *Tbx21* and *Ifng* expression during Th1 polarization. At the early stage of Th1 differentiation, *Ifng* expression was significantly higher at 12 and 24 h, whereas *Tbx21* was higher at 24 h but not at 12 h in *Abcd1*-deficient CD4^+^ T cells compared with wild-type cells (**Figure 3B**). Intracellular staining showed that both the percentage and mean fluorescence intensity (MFI) of IFN-γ–producing cells were significantly higher in *Abcd1*-deficient CD4^+^ T cells compared with wild-type CD4^+^ T cells (**Figure 3C**). These results indicate that *Abcd1* deficiency promotes the differentiation of IFN-γ–producing Th1-type cells, consistent with the enhanced Th1-type responses in antigen-immunized *Abcd1*-deficient mice (**Figure 1**).

### Decreased Blimp-1 expression in *Abcd1*-deficient CD4^+^ T cells

**Figure 4:**
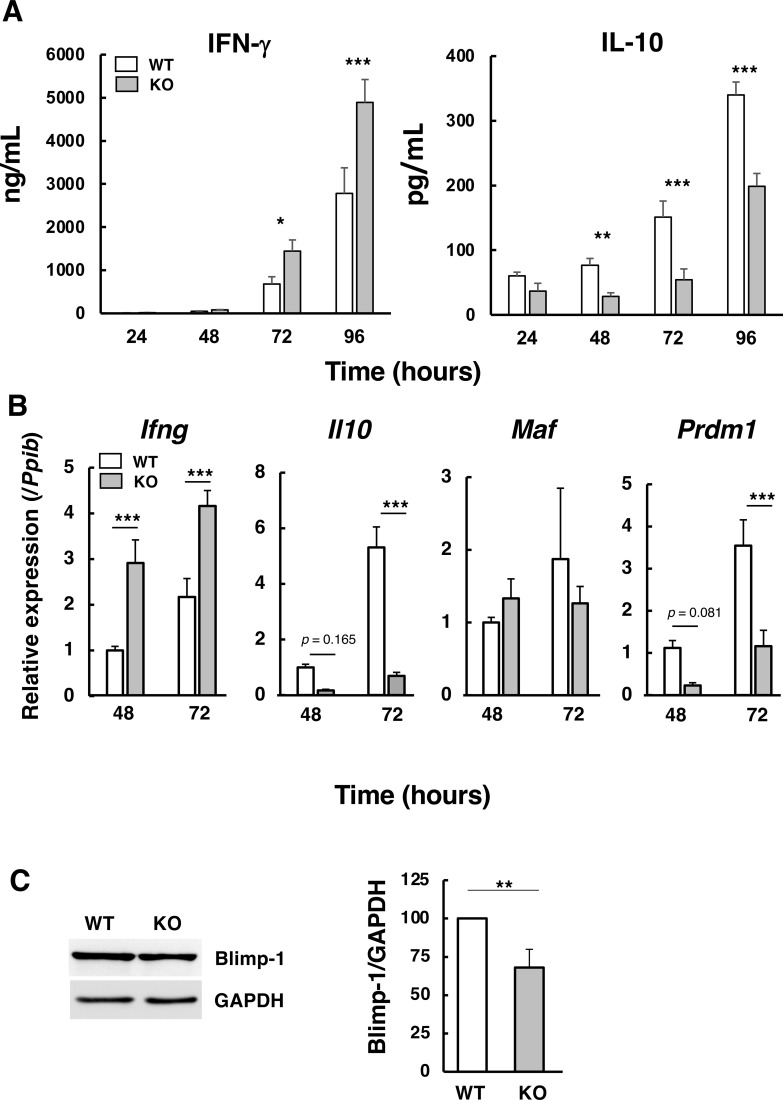
IFN-γ and IL-10 production in *Abcd1*-deficient CD4^+^ T cells during Th1 differentiation. **(A)** Naïve CD4^+^ T cells were activated with anti-CD3/anti-CD28 antibodies in the presence of IL-12 and a neutralizing anti-IL-4 antibody (Th1 polarizing conditions). At 24, 48, 72, and 96 h, culture supernatants from wild-type (WT; white bars) and *Abcd1*-deficient (KO; gray bars) cells were collected and IFN-γ and IL-10 were quantified by ELISA. Data are mean ± SD (n = 5). *p < 0.05, **p < 0.01, ***p < 0.001 compared with WT (two-way ANOVA). **(B)** RT-qPCR of *Ifng* and *Il10* was performed 48 h and 72 h after stimulation as in **(A)**. Expression levels were normalized to *Ppib* and expressed relative to WT at 0 h (set to 1). Data are mean ± SD (n = 5). ***p < 0.001 compared with WT (two-way ANOVA). **(C)** Immunoblotting of Blimp-1 was performed 72 h after stimulation. Equal amounts of protein (50 µg per lane) were separated by SDS PAGE and probed with anti-Blimp-1 and anti-GAPDH antibodies. Band intensities were quantified using LAS 4000 and ImageJ. Blimp-1 signals were normalized to GAPDH and expressed relative to WT (set to 100); KO values are shown as a percentage of WT. ** *p* < 0.01 compared with WT (Student’s *t*-test).

During Th1 differentiation *in vitro*, IFN-γ and IL-10 levels increased over time in CD4^+^ T cells from both wild-type and *Abcd1*-deficient mice (**Figure 4A**). Because IL-10 is co-produced with IFN-γ by Th1 cells to restrain inflammation (31), we hypothesized that *Abcd1* deficiency might alter IL-10 regulation. Indeed, *Ifng* and *Il10* expression levels were reciprocally regulated in differentiating *Abcd1*-deficient CD4^+^ T cells compared with wild-type CD4^+^ T cells (**Figures 4A, B**). We next focused on *Prdm1*, encoding the transcriptional repressor Blimp-1, because it represses T-bet and IFN-γ and is required for the emergence of IL-10–producing effector T cells; therefore, altered *Prdm1* provides a mechanistic link between elevated IFN-γ and reduced IL-10. Notably, *Prdm1* mRNA showed a decreasing trend at 48 h (n.s., *p* = 0.081) and was significantly reduced at 72 h in *Abcd1*-deficient CD4^+^ T cells compared with wild-type cells (**Figure 4B**). Consistently, Blimp1 protein levels were significantly lower at 72 h (**Figure 4C**).

Given that Blimp-1 represses IFN-γ and T-bet and is essential for IL-10-producing cells (32, 33), its reduction likely promotes T-bet induction and IFN-γ upregulation while impairing IL-10 expression. In contrast, *Maf* (c-Maf), a key IL-10 regulator, remained unchanged (**Figure 4B**). Therefore, decreased Blimp-1 expression may underlie the Th1 bias in *Abcd1*-deficient CD4^+^ T cells.

### Increased LXR activation in *Abcd1*-deficicnt CD4^+^ T cells

**Figure 5:**
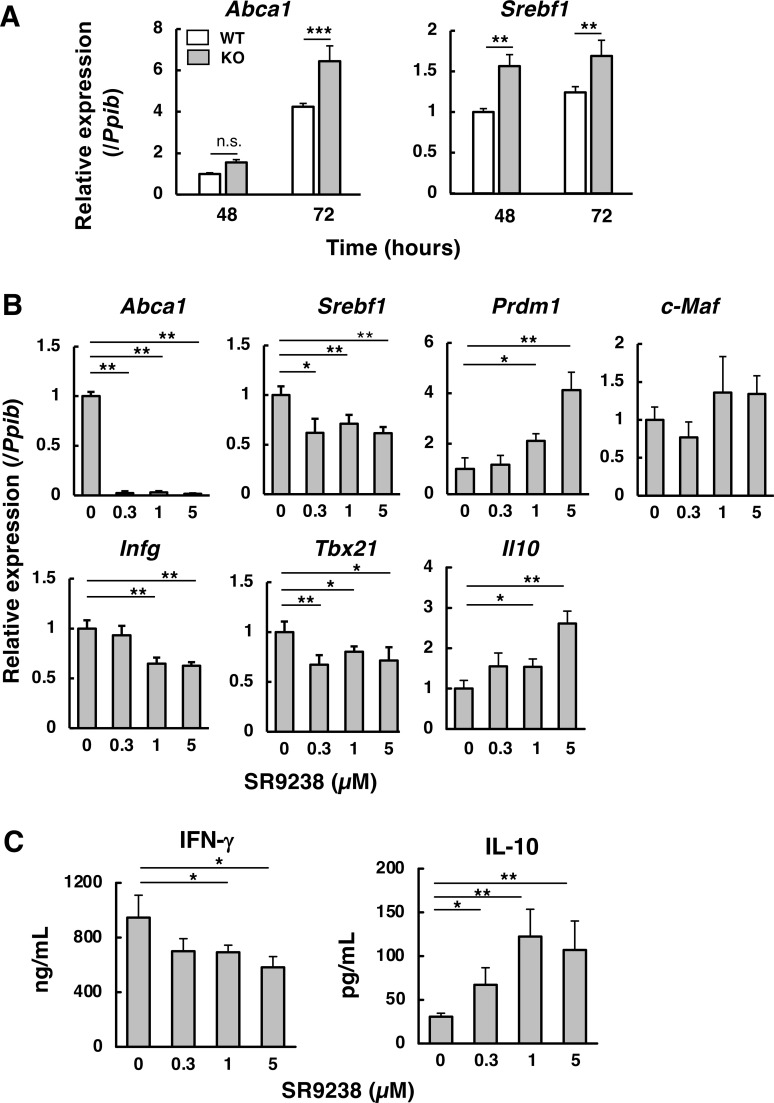
LXR antagonism decreases Th1-type responses in *Abcd1*-deficient CD4^+^ T cells. **(A)** *Abca1* and *Srebf1* mRNA levels in CD4^+^ T cells from wild-type (WT; white bars) and *Abcd1*-deficient (KO; gray bars) mice were quantified by RT-qPCR as in **Figure 4**. Expression was normalized to *Ppib* and expressed relative to WT at 48 h (set to 1). Data are mean ± SD (n =3). **p < 0.01, ***p < 0.001 compared with WT (two-way ANOVA). **(B)** Naïve CD4^+^ T cells from *Abcd1*-deficient mice were activated as above; after 48 h, the medium was replaced with the same medium containing SR9238 (0, 0.3, 1.0, or 5.0 µM) and cells were cultured for an additional 24 h. Gene expression was quantified by RT-qPCR, normalized to *Ppib*, and expressed relative to 0 µM (set to 1). Data are mean ± SD (n =4). Statistical significance was determined using one-way ANOVA followed by Dunnett’s multiple comparison test using 0 µM as the control: *p < 0.05, **p< 0.01 vs 0 µM. **(C)** IFN-γ and IL-10 concentrations in culture supernatants were quantified by ELISA after SR9238 treatment as in **(B)**. Bars: white, WT; gray, KO (panel A). Data are mean ± SD (n = 4). Statistical significance was determined using one-way ANOVA followed by Dunnett’s multiple comparison test using 0 µM as the control. *p < 0.05, **p< 0.01 vs 0 µM.

It has been reported that Blimp-1 expression is suppressed by activation of LXR, a transcriptional regulator of cholesterol metabolism (34). Therefore, we next investigated whether LXR activation contributes to the decrease in Blimp-1 expression in *Abcd1*-deficient CD4^+^ T cells. Under Th1-polarizing conditions, expression of the LXR target gene *Srebf1* was significantly higher in *Abcd1*-deficient CD4^+^ T cells than in wild-type cells at both 48 h and 72 h (**Figure 5A**). In contrast, *Abca1* showed a more modest, time-dependent increase—reaching statistical significance at 72 h but not at 48 h, possibly reflecting lower basal *Abca1* expression at the earlier time point. Together, these results suggest enhanced LXR signaling activity in *Abcd1*-deficient CD4^+^ T cells during Th1 differentiation.

When *Abcd1*-deficient CD4^+^ T cells were incubated with the LXR antagonist SR9238, the expression of *Abca1* and *Srebf1* was decreased, confirming that LXR signaling was downregulated by SR9238 (**Figure 5B**). In the presence of SR9238, *Prdm1* (Blimp-1) expression was upregulated, whereas *Maf* was not (**Figure 5B**). In addition, the expression of *Ifng* and *Tbx21* (T-bet) and the production of IFN-γ were significantly decreased (**Figures 5B, C**). In contrast, *Il10* expression and IL-10 production were increased by SR9238 treatment (**Figures 5B, C**). These results consistently indicate that activated LXR in *Abcd1*-deficient CD4^+^ T cells suppresses Blimp-1 expression, resulting in increased IFN-γ production and decreased IL-10 production.

**Figure 6:**
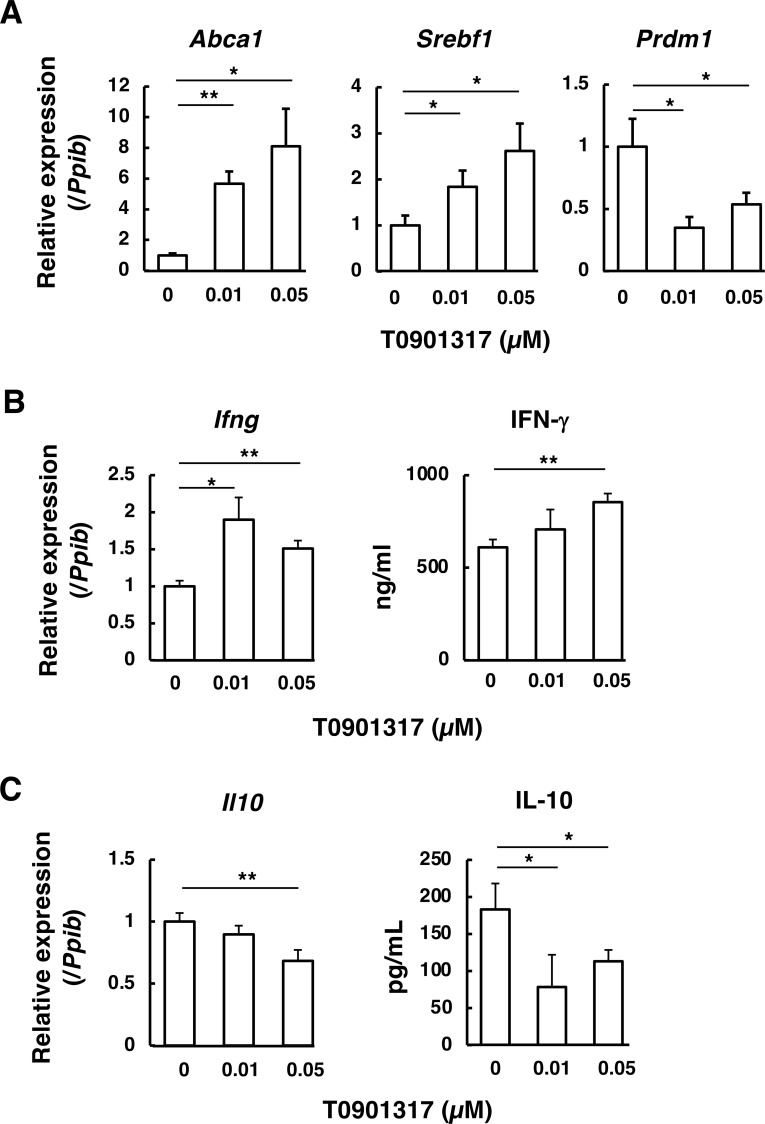
Effect of the LXR agonist T0901317 on Th1-type responses in wild-type CD4^+^ T cells. Naïve wild-type CD4^+^ T cells were activated under Th1-polarizing conditions (IL-12 plus a neutralizing anti-IL-4 antibody), as in **Figure 5**. After 48 h, the medium was replaced with the same medium containing the LXR agonist T0901317 (0, 0.01, or 0.05 µM), and cells were cultured for an additional 24 h. (**A–C)** Gene expression was quantified by RT-qPCR, normalized to *Ppib*, and expressed relative to 0 µM (set to 1). **(B, C)** IFN-γ and IL-10 concentrations in culture supernatants were quantified by ELISA. Data are mean ± SD (n = 4). Statistical significance was determined using one-way ANOVA followed by Dunnett’s multiple comparison test using 0 µM as the control.: *p < 0.05, **p< 0.01 vs 0 µM.

In contrast, when T0901317, a high-affinity ligand for LXR, was added to wild-type CD4^+^ T cells during Th1 differentiation, *Prdm1* (Blimp-1) expression was markedly repressed, accompanied by increased expression of *Abca1* and *Srebf1* (**Figure 6A**). The expression of *Ifng* and the production of IFN-γ were increased by T0901317 treatment (**Figure 6B**). Concurrently, *Il10* expression and IL-10 production were significantly decreased (**Figure 6C**). These results support the conclusion that LXR activation increases IFN-γ production and decreases IL-10 production through suppression of Blimp-1.

### Increased production of 25-hydroxycholesterol by *Abcd1*-deficient CD4^+^ T cells

**Figure 7:**
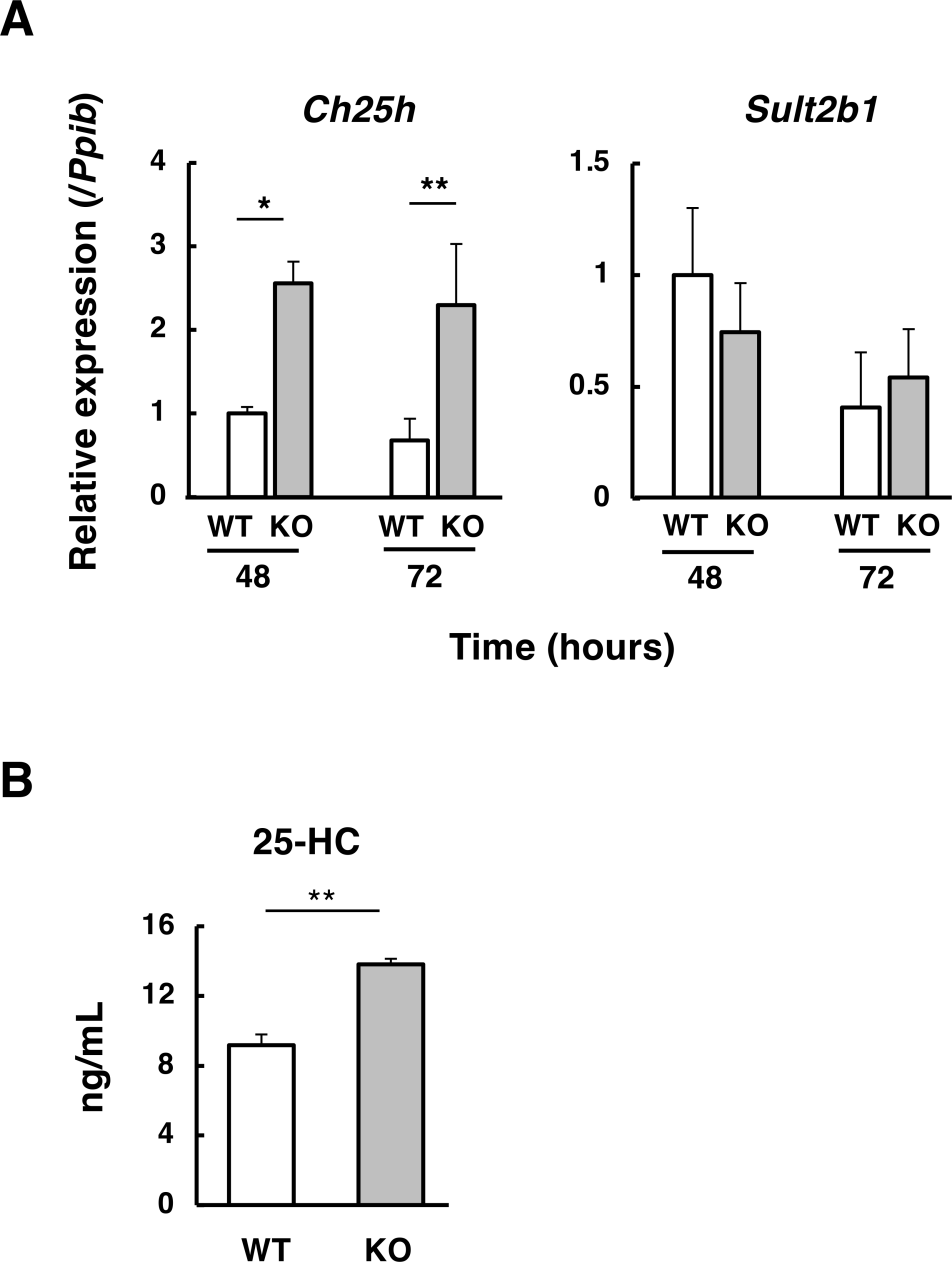
*Ch25h* expression and 25-hydroxycholesterol production in *Abcd1*-deficient CD4^+^ T cells. **(A)** Naïve CD4^+^ T cells were isolated from wild-type (WT; white bars) and *Abcd1*-deficient (KO; gray bars) mice, activated for 48 h, and *Ch25h* mRNA was quantified by RT-qPCR. Expression levels were normalized to *Ppib* and expressed relative to WT (set to 1). *p < 0.05, **p < 0.01 compared with WT (two-way ANOVA). **(B)** 25-Hydroxycholesterol (25-HC) concentrations in culture supernatants were quantified by gas chromatography-mass spectrometry (GC-MS) 72 h after stimulation. Data are mean ± SD (n = 4). **p < 0.01 compared with WT (Student’s *t*-test).

An endogenous ligand for LXR has not yet been definitively identified, but oxysterols are strong candidates (35, 36). Interestingly, we found that *Ch25h*, which encodes cholesterol 25-hydroxylase (CH25H)—an enzyme that produces 25-hydroxycholesterol (25-HC)— was significantly upregulated in *Abcd1*-deficient CD4^+^ T cells under Th1-polarizing conditions (**Figure 7A**). In contrast, the expression of *Sult2b1*, which encodes an oxysterol-metabolizing enzyme that converts 25-HC to 25-hydroxycholesterol 3-sulfate (25-HC3S), did not differ between the two groups. Since cholesterol 25-hydroxylase converts cholesterol to 25-HC, we next examined whether the level of 25-HC was higher in *Abcd1*-deficient CD4^+^ T cells than in wild-type CD4^+^ T cells. Accordingly, the level of 25-HC in the culture supernatants of *Abcd1*-deficient CD4^+^ T cells (13 ng/mL) was significantly higher than that in wild-type CD4^+^ T cells (9 ng/mL) (**Figure 7B**). These results suggest that increased 25-HC may be associated with enhanced LXR signaling activity in *Abcd1*-deficient CD4^+^ T cells.

**Figure 8:**
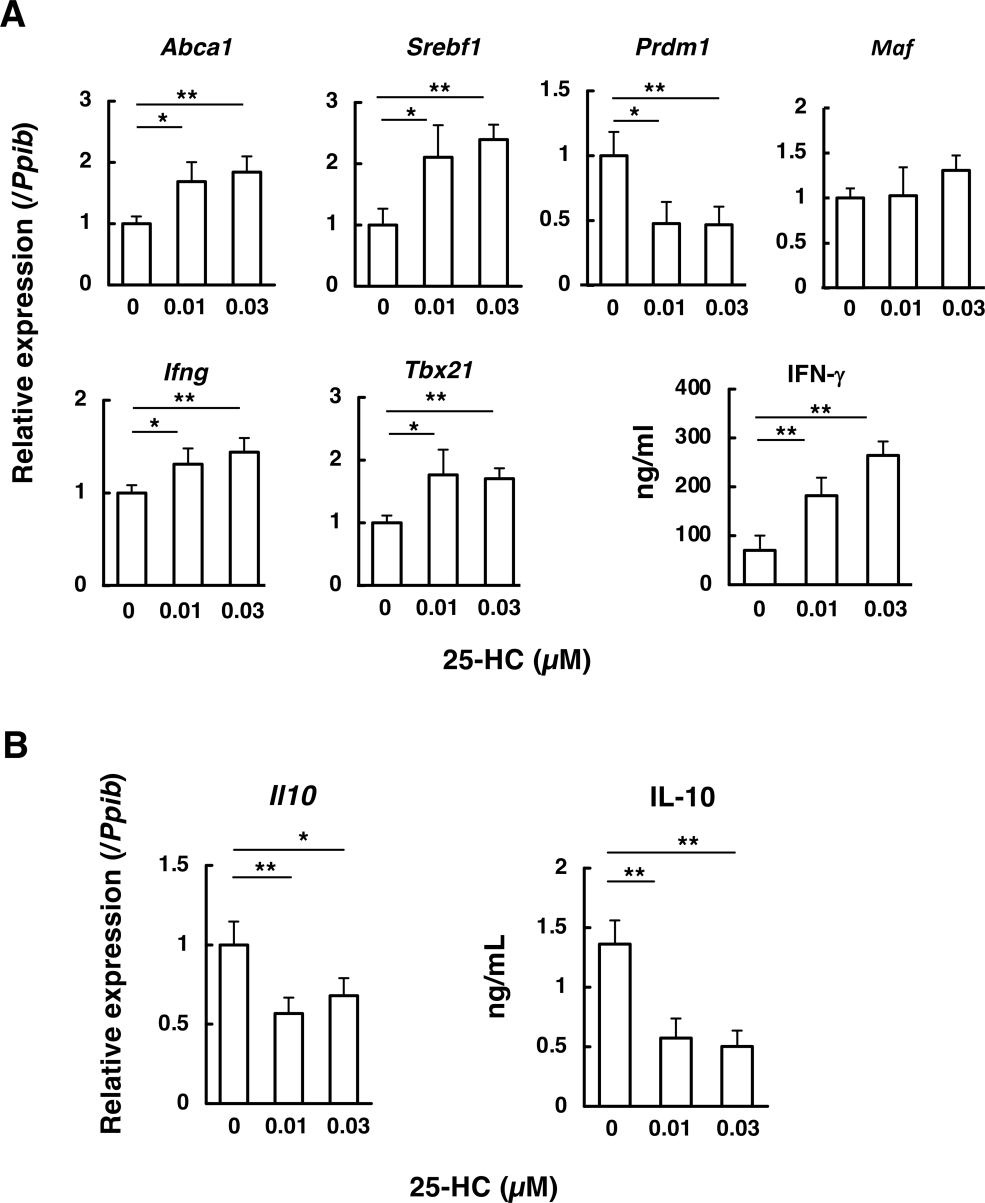
Effect of 25-hydroxycholesterol on gene expression and IFN-γ/IL-10 production in wild-type CD4^+^ T cells. Naïve wild-type (WT) CD4^+^ T cells were activated under Th1-polarizing conditions (anti-CD3/anti-CD28 antibodies, IL-12, IL-2, and a neutralizing anti-IL-4 antibody) in the presence of 25-hydroxycholesterol (25-HC) at final concentrations of 0, 0.01, or 0.03 µM. After 48 h, gene expression was quantified by RT-qPCR **(A, B)**, normalized to *Ppib*, and expressed relative to 0 µM (set to 1). IFN-γ and IL-10 concentrations in culture supernatants were quantified by ELISA **(A, B)**. Data are mean ± SD (n = 6). Statistical significance was determined using one-way ANOVA followed by Dunnett’s multiple comparison test using 0 µM as the control: *p < 0.05, **p< 0.01 vs 0 µM.

Next, we examined whether physiological concentrations of 25-HC could mimic the phenotypes observed in *Abcd1*-deficient CD4^+^ T cells. Because 25-HC appeared to affect CD4^+^ T cell viability, this experiment was conducted in the presence of IL-2 to mitigate this negative effect. 25-HC minimally affected Th1 cell viability at approximately 0.03 µM (**Supplementary Figure 4**). When wild-type CD4^+^ T cells were treated with this concentration of 25-HC, LXR-targeted genes (*Srebf1* and *Abca1*) were upregulated, indicating that 25-HC activates LXR signaling (**Figures 8A, upper**). Concurrently, 25-HC significantly enhanced the expression of *Ifng* and *Tbx21* and the production of IFN-γ (**Figures 8A, lower**). In contrast, 25-HC inhibited *Prdm1* (Blimp-1) expression without altering *Maf* expression (**Figures 8A, upper**). In addition, 25-HC suppressed *Il10* expression and IL-10 production (**Figure 8B**). Overall, the response induced by 25-HC in wild-type CD4^+^ T cells closely resembled the molecular expression phenotypes observed in *Abcd1*-deficient CD4^+^ T cells.

**Figure 9:**
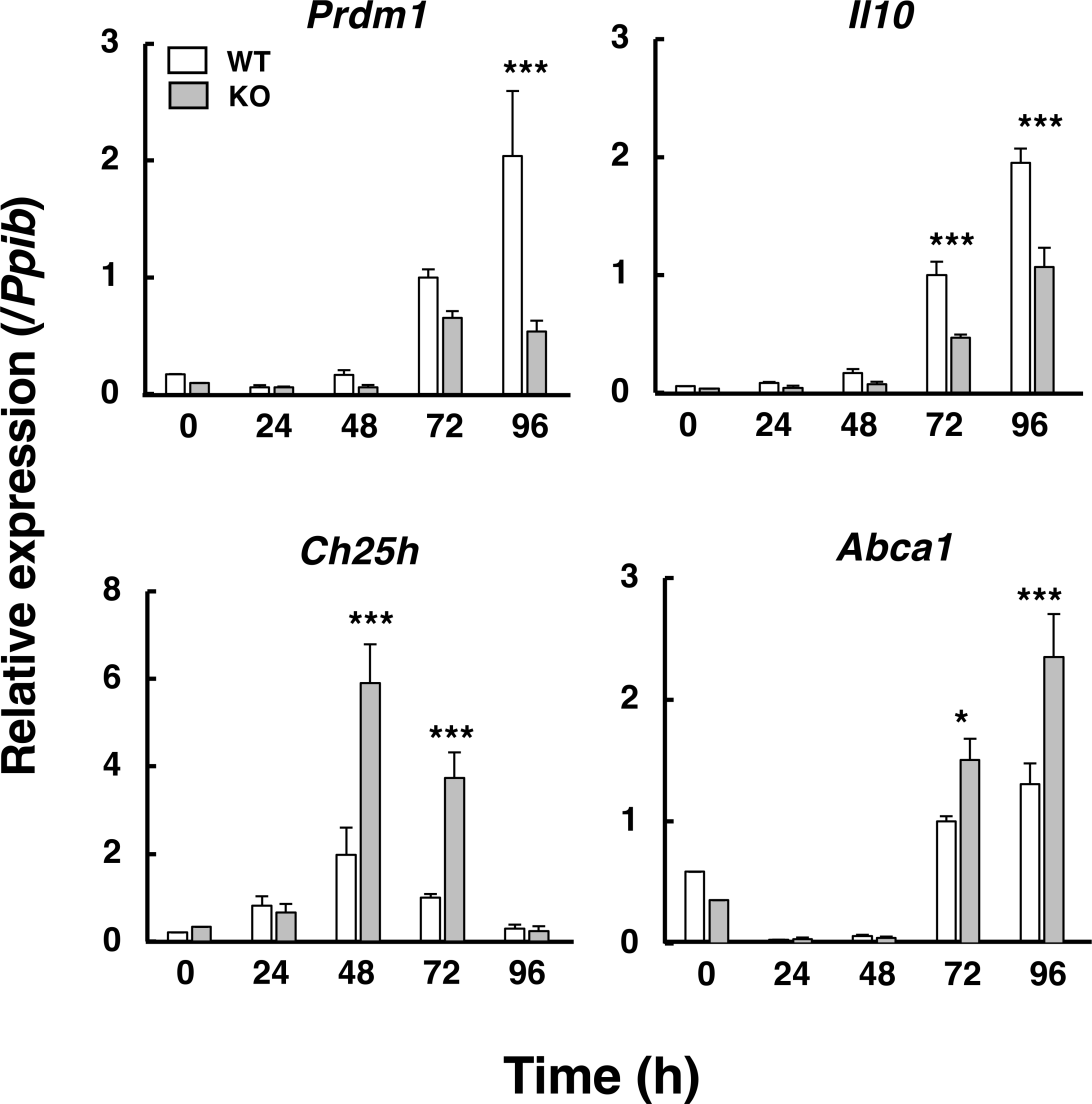
Sequential expression of *Ch25h* and *Prdm1* regulates cytokine programs in *Abcd1*-deficient CD4^+^ T Cells. Time course RT-qPCR of *Prdm1*, *Ch25h, Il10*, and *Abca1* in wild-type (WT; white bars) and *Abcd1*-deficient (KO; gray bars) CD4^+^ T cells at 0, 24, 48, 72, and 96 h after stimulation under Th1-polarizing conditions. Expression levels were normalized to *Ppib* and expressed relative to WT at 72 h (set to 1). Data are mean ± SD (n = 6). *p < 0.05, ***p < 0.001 compared with WT at the same time point (two-way ANOVA).

Temporal expression of *Ch25h* and *Prdm1* in differentiating naïve CD4^+^ T cells may regulate IFN-γ and IL-10 programs during Th1-lineage commitment. During Th1 differentiation, *Ch25h* expression peaked at 48 h, then gradually decreased and disappeared by 96 h (**Figure 9**). In *Abcd1*-deficient CD4^+^ T cells, *Ch25h* expression was significantly higher than in wild-type CD4^+^ T cells at 48 and 72 h. In contrast, *Prdm1* (Blimp-1) expression increased later, rising at 72 h and remaining stable or becoming repressed by 96 h. *Il10* and *Abca1* expression levels were elevated after 72 h. Therefore, increased *Ch25h* expression precedes *Prdm1* repression, resulting in enhanced IFN-γ and reduced IL-10 production during late Th1 differentiation in *Abcd1*-deficient CD4^+^ T cells. These findings support the idea that an initial burst of 25-HC produced by CH25H activates LXR and subsequently downregulates Blimp-1.

## Discussion

T cells are increasingly recognized as important contributors to demyelinating diseases; however, their role in X-ALD remains incompletely understood. In the present study, we focused on defining cell-intrinsic alterations in CD4^+^ T cells caused by Abcd1 deficiency. Although the *Abcd1*-deficient mouse does not develop the cerebral inflammatory demyelination characteristic of CALD, this model provides a valuable system to examine T cell–specific consequences of Abcd1 loss independent of overt CNS pathology.

Here, we show that *Abcd1*-deficient CD4^+^ T cells exhibit a Th1-skewed phenotype, characterized by *Ifng* and *Tbx21* upregulation and increased IFN-γ production under antigen-specific immunization *in vivo* and Th1-polarizing conditions *in vitro*. Collectively, these results indicate that *Abcd1* deficiency intrinsically enhances Th1 differentiation in CD4^+^ T cells and suggest a cell-intrinsic role for Abcd1 in restraining pro-inflammatory T cell programming relevant to X-ALD pathogenesis.

Mechanistically, we identified LXR signaling as a key regulator of this cytokine imbalance. In *Abcd1*-deficient CD4^+^ T cells, the LXR pathway is activated; pharmacological antagonism using SR9238 restored *Prdm1* (Blimp-1) and IL-10 while reducing IFN-γ, whereas LXR activation with T0901317 in wild-type cells recapitulated the *Abcd1*-deficient phenotype. This is consistent with prior reports that LXR activation constrains Blimp-1 and limits IL-10 in Tr1 cells (34). Notably, *Prdm1* expression is markedly suppressed at later stages of Th1 differentiation in *Abcd1*-deficient cells, coinciding with increased *Ifng* and decreased *Il10*, suggesting that reduced Blimp-1 accounts for both IFN-γ de-repression and diminished IL-10 during late Th1 differentiation.

25-Hydroxycholesterol (25-HC) functions as an LXR agonist and fine-tunes CD4^+^ T cell polarization toward proinflammatory states (37). Consistent with observations in *Abcd1*-deficient mouse brain, ALD patient fibroblasts, and *Abcd1*-deficient BV-2 microglial-like cell lines (21, 38, 39), we observed increased *Ch25h* expression during Th1 differentiation in *Abcd1*-deficient CD4^+^ T cells and higher 25-HC levels in culture supernatants. Importantly, exposing wild-type CD4^+^ T cells to a physiological concentration of 25-HC (10 ng/mL; within the 2–30 ng/mL plasma range reported in humans and mice) (40) induced an *Abcd1*-deficiency-like phenotype—decreased *Prdm1*, increased IFN-γ, and decreased IL-10. In our system, 25-HC suppressed *Prdm1* without affecting *Maf* (c-Maf), whereas human Th1 cells have been reported to downregulate *MAF* to limit IL-10 (41), suggesting species- and context-dependent regulation. Additionally, we found that increased *Ch25h* expression precedes *Prdm1* suppression during Th1 differentiation, supporting a temporal link between 25-HC production and Blimp-1 downregulation. This observation further strengthens the mechanistic model in which 25-HC–LXR signaling promotes Th1 polarization by repressing *Prdm1*, thereby enhancing IFN-γ and reducing IL-10 production.

How *Abcd1* deficiency triggers a CH25H-dependent surge in 25-HC remains unresolved. Based on prior links between VLCFA load, oxidative stress, and mitochondrial reactive oxygen species (ROS) (9, 42), we speculate that VLCFA accumulation—a hallmark of ALD—may increase oxidative stress and mitochondrial ROS, which could, in antigen-activated T cells, engage NFAT signaling to enhance IFN-γ production (43, 44). Given that CH25H is an interferon-stimulated gene potentially inducible by IFN-γ (45), we tentatively propose a working model in which a VLCFA–ROS–IFN-γ–CH25H axis operates upstream of the 25-HC–LXR–Blimp-1 pathway to reinforce Th1 polarization in *Abcd1*-deficient CD4^+^ T cells. We emphasize that these upstream steps are inferred rather than demonstrated in the current study, and will require direct testing (e.g., ROS quantification, NFAT activity, and IFN-γ–dependent CH25H induction in *Abcd1*-deficient CD4^+^ T cells). Alternatively, other pathways may likewise converge on CH25H induction and LXR activation—such as type I interferon–driven programs, TLR-dependent innate signaling, or glial- and endothelial-derived inflammatory cues—underscoring that multiple inputs could produce the observed 25-HC–LXR–Blimp-1 axis (46).

Alternative non–T-cell–centric mechanisms are likely to shape the cerebral inflammatory milieu in X-ALD, including brain endothelial dysfunction with BBB breakdown, innate glial activation, and astrocyte-driven toxicity (16, 17, 23, 47). Consistent with this view, our data support a predominantly permissive/amplifying role for *ABCD1*-deficient CD4^+^ T cells—rather than a primary causal trigger—acting within these vascular and glial contexts. In multiple sclerosis (MS), infiltrating T cells are detected within active demyelinating lesions, whereas in X-ALD, IFN‐γ–producing T cells localize at lesion margins adjacent to microglia and astrocytes (6); Th1-derived effectors regulate neuroinflammatory programs in these glia (47–50). We therefore posit that the enhanced Th1 response of infiltrating *ABCD1*-deficient CD4^+^ T cells could exacerbate and accelerate cerebral inflammation through T cell–glia interactions.

Our mechanistic model integrates pharmacological and transcriptional evidence; however, establishing causality between the proposed VLCFA–ROS–IFN-γ–CH25H axis and the 25-HC–LXR–Blimp-1 pathway will require target perturbations (e.g., CH25H knockdown/knockout, ROS scavenging, NFAT inhibition) in *Abcd1*-deficient T cells *in vivo*. Likewise, cell-type-specific *Abcd1* deletion and adoptive-transfer models will help disentangle T-cell–intrinsic versus microenvironmental contributions and establish the relative roles of peripheral priming versus CNS reactivation.

Our findings suggest that an enhanced Th1-type response in activated CD4^+^ T cells may represent a predisposing immunological state that could contribute to X-ALD-associated inflammation under additional permissive conditions. These insights provide a rationale for therapeutic strategies that modulate CD4^+^ T cell responses—potentially by targeting the 25-HC–LXR–Blimp-1 axis, CH25H activity, or upstream oxidative signaling—to prevent or attenuate neuroinflammation in X-ALD.

## Data Availability

The original contributions presented in the study are included in the article/**Supplementary Material**. Further inquiries can be directed to the corresponding author.

## Supplementary material

Supplementary Figure 1 Flow cytometric analysis of draining lymph node T cell populations after OVA/CFA immunization. Wild-type (WT) and *Abcd1*-deficient (knockout; KO) mice were immunized in the footpads with ovalbumin (OVA) emulsified in complete Freund’s adjuvant (CFA). Two weeks later, cells from draining lymph nodes (dLNs) were collected, resuspended in FACS buffer (0.5–1 × 10^6^ cells/mL), and analyzed by flow cytometry. Immunization via the footpad results in localized activation of draining lymph nodes. Gating: P2, CD4^+^ T cells; P3, CD8^+^ T cells; Q1, effector memory T cells (CD44^high^ CD62L^high^); Q2, central memory T cells (CD44^high^ CD62L^low^); Q4, naive T cells (CD44^low^ CD62L^high^). One representative plot is shown. Graphs summarize the percentages of CD4^+^ and CD8^+^ T cells among total lymphocytes, and the frequencies of naive, central memory, and effector memory CD4^+^ T cell subsets among CD4^+^ T cells. Data are mean ± SD (n = 3 mice per group). n.s., not significant.

Supplementary Figure 2 Proliferation of OVA-restimulated draining lymph node cells. Wild-type (WT) and *Abcd1*-deficient (KO) mice were immunized in the footpads with ovalbumin (OVA) emulsified in complete Freund’s adjuvant (CFA). Two weeks later, cells from draining lymph nodes (dLNs) were cultured *ex vivo* with OVA (0, 0.1, 1, or 10 µM) for 4 days, as in **Figure 1**. This *ex vivo* restimulation assay assesses antigen-specific T-cell reactivity in draining lymph nodes under experimental conditions. Proliferation was assessed by the MTT assay. Bars: white, WT; gray, KO. Data are mean ± SD (n =5). ***p* < 0.01, ****p* < 0.001 compared with WT (two-way ANOVA). n.s., not significant.

Supplementary Figure 3 Flow cytometric analysis of CD4^+^ T cell phenotypes after *ex vivo* activation and IL-2 expansion. Total CD4^+^ T cells were isolated from the spleens of wild-type (WT) and *Abcd1*-deficient (KO) mice, activated with anti-CD3/anti-CD28 antibodies for 72 h, and then cultured in IL-2–containing medium (10 ng/mL) for an additional 72 h. Cells were stained for surface markers (CD4, CD8, CD44, CD62L) and analyzed by flow cytometry. One representative plot is shown. Graphs summarize the percentage of CD4^+^ T cells among total cells (purity) and the frequencies of naive (CD62L^high^ CD44^low^), central memory (CD62L^high^ CD44^high^), and effector memory (CD62L^low^ CD44^high^) subsets among CD4^+^ T cells. Data are mean ± SD (n = 3). n.s., not significant.

Supplementary Figure 4 Effect of 25-hydroxycholesterol on viability of wild-type CD4^+^ T cells. Naïve CD4^+^ T cells were isolated from wild-type (WT) mouse spleens and activated under Th1 polarizing conditions (anti-CD3/anti-CD28 antibodies, IL-12, IL-2, and a neutralizing anti-IL-4 antibody), in the presence of 25-hydroxycholesterol (25-HC) at 0, 0.01, or 0.03 µM, as in **Figure 7**. After 48 h, cell viability was assessed by the MTT assay. Data are mean ± SD (n = 4). Statistical significance was determined using one-way ANOVA followed by Dunnett’s multiple comparison test using 0 µM as the control: **p* < 0.05, ***p* < 0.01.
