# Supplementary material for: Peroxisomal ABCD1 deficiency in mice drives Th1 bias through 25-HC–LXR signaling in CD4+ T cells
Source: Front Immunol. 2026 May 20;17:1722647. doi: 10.3389/fimmu.2026.1722647 (PMC13230122; doi:10.3389/fimmu.2026.1722647)
Supplement: Supplementary file 1 [file Table1.docx]

**Supplemental Table 1**

| Primer | Forward 5’→3’ | Reverse 5’→3’ |
| --- | --- | --- |
| *Ppib* | TGGAGAGCACCAAGACAGACA | TGCCGGAGTCGACAATGAT |
| *Ifng* | GCCACGGCACAGTCATTGA | TGCTGATGGCCTGATTGTCTT |
| *Il10* | TGCTCCTAGAGCTGCGGACT | AGGCTTGGCAACCCAAGTAACC |
| *Prdm1* | TCAAGCCGAGGCATCCTTAC | CCTCTCTGGAATAGATCCGCC |
| *Tbx21* | GGTTGGAGGTGTCTGGGAAGC | GCCACGGTGAAGGACAGGAAT |
| *Maf* | AGCAGTTGGTGACCATGTCG | TGGAGATCTCCTGCTTGAGG |
| *Abca1* | GTGTGAGCAAAGCCAAGCA | CGACCACCCATACAGCAAGA |
| *Srebf1* | GTCAAAACCAGCCTCCCAAG | CAGTCCCCGTCCACAAAGA |
| *Ch25h* | CGTGAAAGGTAAAGATGGTGAGTG | ATCTGCCTGCTGCTCTTCG |
| *Sult2b1* | GGTCAAACCAGGAGCCAAAC | GCTCTCACCTCCCTATTGAACTCT |
