# Supplementary figures and images for: Peroxisomal ABCD1 deficiency in mice drives Th1 bias through 25-HC–LXR signaling in CD4+ T cells
Source: Front Immunol. 2026 May 20;17:1722647. doi: 10.3389/fimmu.2026.1722647 (PMC13230122; doi:10.3389/fimmu.2026.1722647)

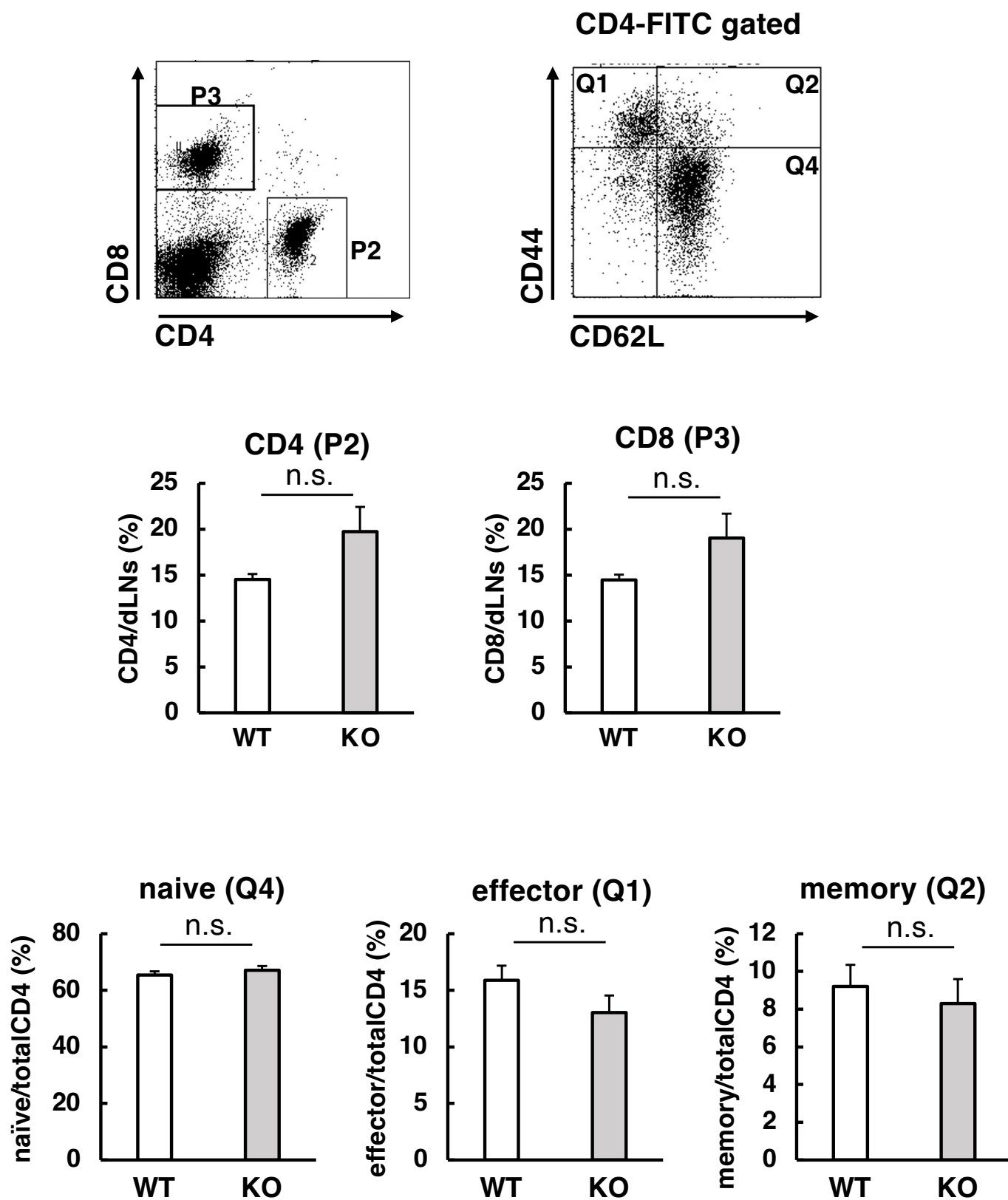

Suppl. Fig. 1

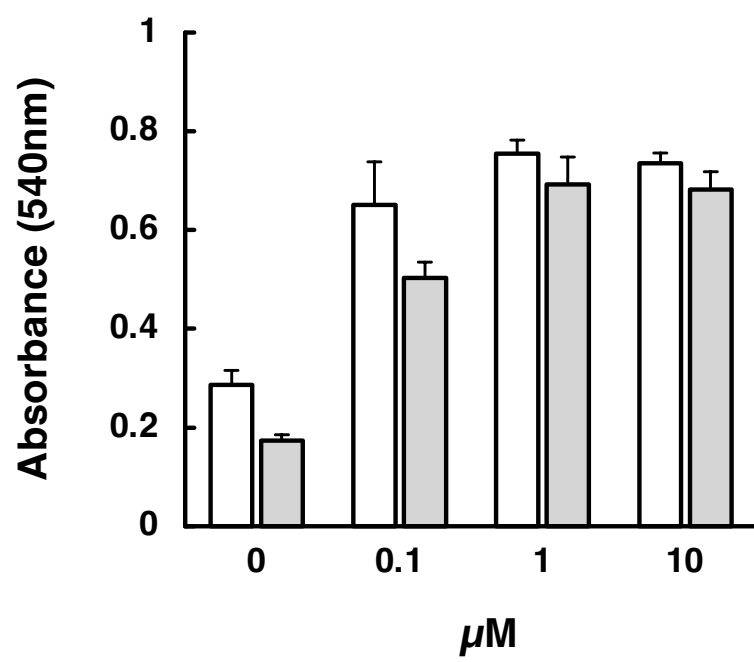

## CD4-FITC gated

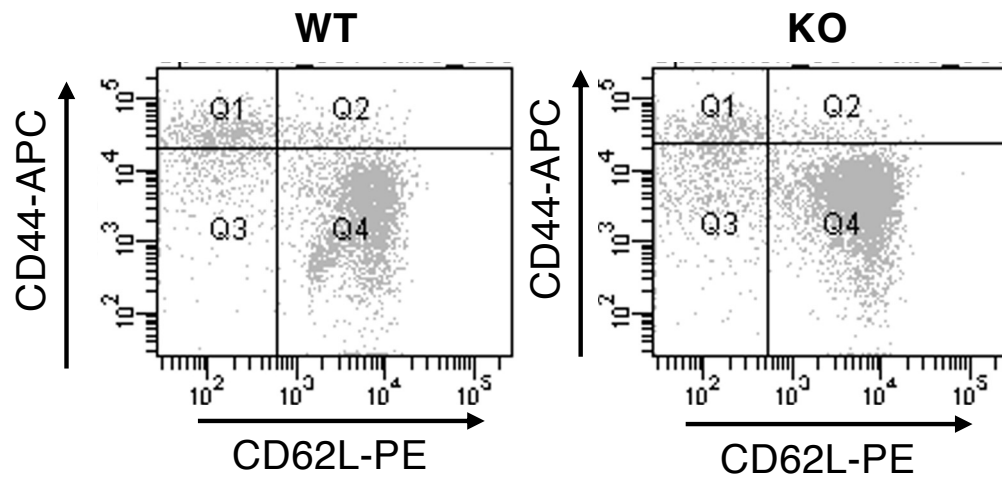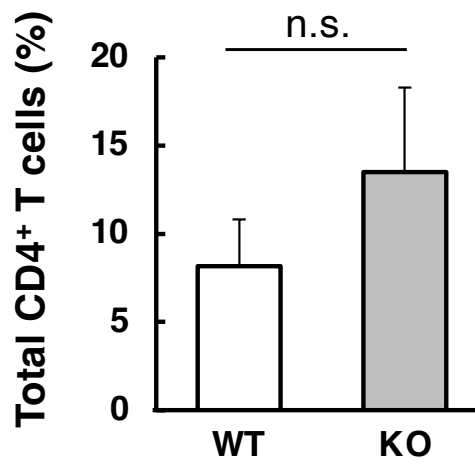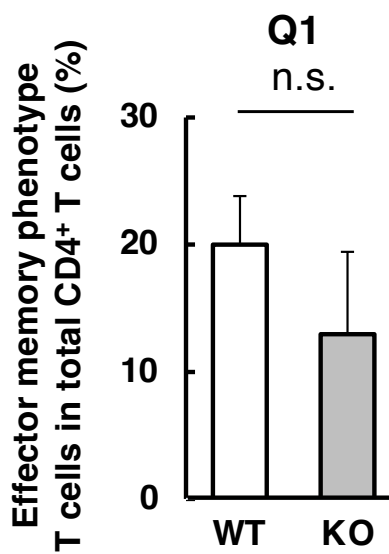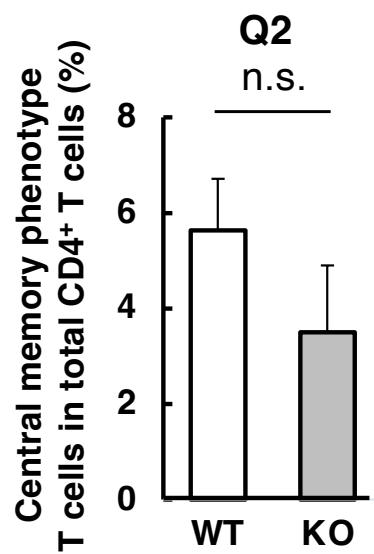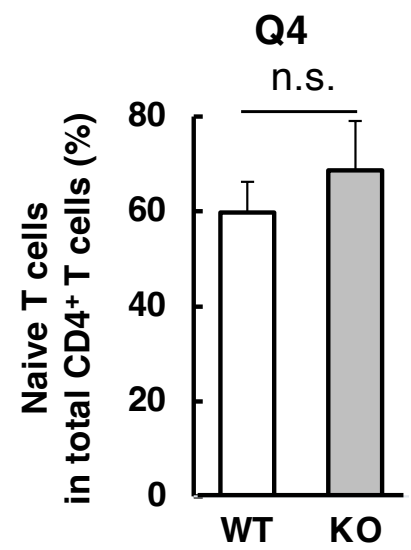

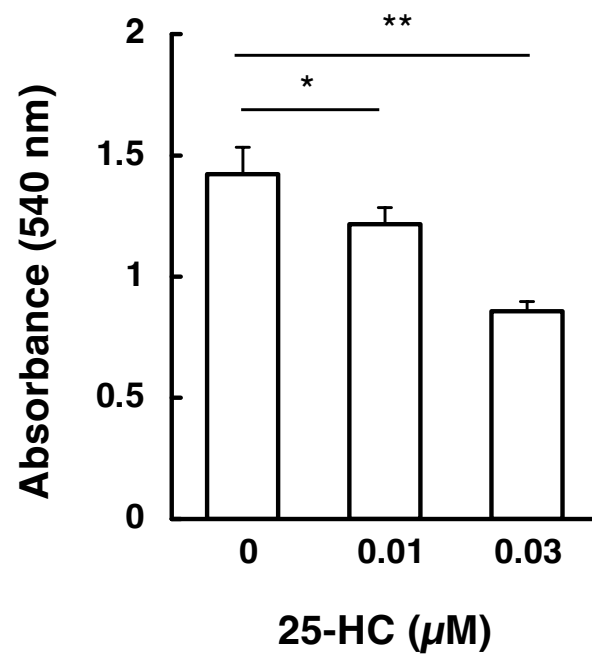

Supplement: Supplementary Figure 1 — Flow cytometric analysis of draining lymph node T cell populations after OVA/CFA immunization. Wild-type (WT) and Abcd1-deficient (knockout; KO) mice were immunized in the footpads with ovalbumin (OVA) emulsified in complete Freund’s adjuvant (CFA). Two weeks later, cells from draining lymph nodes (dLNs) were collected, resuspended in FACS buffer (0.5–1 × 10^6^ cells/mL), and analyzed by flow cytometry. Immunization via the footpad results in localized activation of draining lymph nodes. Gating: P2, CD4+ T cells; P3, CD8+ T cells; Q1, effector memory T cells (CD44high CD62Lhigh); Q2, central memory T cells (CD44high CD62Llow); Q4, naive T cells (CD44low CD62Lhigh). One representative plot is shown. Graphs summarize the percentages of CD4+ and CD8+ T cells among total lymphocytes, and the frequencies of naive, central memory, and effector memory CD4+ T cell subsets among CD4+ T cells. Data are mean ± SD (n = 3 mice per group). n.s., not significant. [file DataSheet1.pdf]
